# Re-negotiating agency – patients using comics to reflect upon acting in situations of abuse in health care

**DOI:** 10.1186/s12913-019-3902-y

**Published:** 2019-01-23

**Authors:** A. Jelmer Brüggemann, Camilla Forsberg, Robert Thornberg

**Affiliations:** 1 Gender and Medicine, Department of Clinical and Experimental Medicine, Linköping University, 581 83 Linköping, Sweden; 2 Department of Thematic Studies – Technology and Social Change, Linköping University, 581 83 Linköping, Sweden; 3 Department of Behavioural Sciences and Learning, Linköping University, 581 83 Linköping, Sweden

**Keywords:** Patient agency, Abuse in health care, Constructivist grounded theory, Comics, Sweden

## Abstract

**Background:**

There is a growing body of international research that displays the prevalence and character of abuse in health care. Even though most of these studies are conducted from a patient perspective little is known about how patients conceptualize their agency in relation to such situations. This study aimed to explore how patients reason about their potential to act in abusive situations.

**Methods:**

Qualitative interviews were conducted with thirteen patients in Sweden. Central in the interviews were three comics, inspired by Boal’s Forum Theatre and part of an earlier online intervention study in which the informants had participated. Each comic showed a situation in which a patient feels abused, and on the opposite side were suggestions for how the patient could act in response. Informants were asked to reflect about situations of abuse and in specific upon the comics. We used the methodology of constructivist grounded theory throughout the study, including the analysis.

**Results:**

It appeared that the informants constantly re-negotiated their and other patients’ agency in relation to the specifics of the event, patients’ and staff’s responsibilities, and the patients’ needs and values. This process questions views of agency as fixed and self-evident, and can be understood as part of changing discourses about patients’ social role and possibilities to organize their care. Using a feminist theory of power we expected the informants to elicit instances of *resistance to* domination, which is central to the comics. While doing that, the informants also hinted at parallel stories of empowerment and less visible forms of agency *in spite of* domination.

**Conclusion:**

The current analysis showed different ways in which the informants constantly re-negotiated their agency in potentially abusive situations. Not only did the informants engage in reflections about immediate responses to these untoward situations, they also engaged in thoughts about strategies that could protect them and counteract abuse in health care over the long-term. This opens up for future research into ways patients organize their care and identify threats and barriers to the care they need, which could be valuable knowledge for care quality improvement.

## Background

This study explores how patients conceptualize their agency in relation to situations of abuse in health care. Agency refers to individual autonomy and the capacity to make own free choices, to self-governing one’s behavior, and to reflect upon oneself and one’s actions, as constituted within social relations and intertwined with the construction of identity [1, 2]. Although agency is associated with freedom, no one is totally independent and free. Bandura argues that freedom is not just the absence of constraints, but also the active selection and production of life circumstances [3]. What needs and possibilities to act do patients see? How do they position other patients, health care professionals, and themselves as patients? Answers to such questions cannot only tell us more about agency in confined action spaces, but also what it means being a patient in Swedish health care settings.

### Abuse in health care

Abuse in health care has been studied in different cultural settings around the globe, for example, in the Nordic countries [4, 5], the UK [6], Peru and Brazil [7], the US [8], and South-Africa [9, 10]. Despite the different settings and antecedents of the abuse, patients’ experiences of dehumanization and neglect appear to be quite similar. The prevalence of abuse is estimated to range between 13 and 30% in female patient populations in northern European countries [5, 11], compared to 8% in Swedish male patients [4]. In all these studies, abuse is described from the patients’ perspective and their suffering, and it is emphasized that staff need not have an intention to harm [12–14]. Experiences of abuse in health care have been described by female patients as a feeling of *being nullified* [15], while male patients felt *mentally pinioned* [16]. Others have described similar incidents as *personal identity threats* attacking patients’ self-worth [6], or in terms of health care providers’ failure to respect patients’ humanity and personhood [17].

At present, little is known about how patients respond in instances where they experience abuse by health care staff, but a recurrent pattern is that patients seem to remain silent toward the health care system and seldom express their experiences to staff [10, 16, 18–20]. Fear and feelings of powerlessness have been identified as strong inhibitors to voicing such feelings and concerns in cases of untoward experiences in health settings [18]. There are no studies that have examined how patients think of their agency in abusive situations.

The current study aims to explore how patients reason about their potential to act in abusive situations while reflecting upon three comics during an interview situation.

### Three comics

The comics were part of a patient intervention that aimed to see if ways could be found to increase patients’ perceived ability to act in situations in which they risk experiencing abuse in health care. In a quantitative evaluation, it was found that the intervention increased patients’ self-estimated ability to see opportunities to act in a given text scenario [21]. The current study was designed as an in-depth follow-up on this intervention with a number of participants.

Comics are increasingly seen as an appropriate and including medium for medical education and educating patients, one reason being its ability to package complex non-verbal information [22]. Developing the comics built on these assumptions, combined with the project’s overall inspiration through Augusto Boal’s Forum Theatre and Theatre of the Oppressed [23, 24]. This pedagogy aims not to provide a behavioral manual or to memorize solutions to specific problems, but rather aims to visualize social structures and power relationships, and equip people with tools to facilitate change [25].

In collaboration with a feminist activist artist and a drama instructor specialized in Boal’s Forum Theatre and critical pedagogy, three comics were developed [21]. Each comic comprised two parts. The first part consisted of five-six frames showing a health care situation that ended up with a patient feeling devastated or abused. This story was followed by the question, ‘What opportunities to act do you have as a patient?’ The second part consisted of another five-six frames that showed different opportunities, each opportunity labelled with a verb followed by a question mark, including questioning, using body language, involving other staff, expressing one’s feelings, confronting afterwards, and leaving the room. These opportunities were followed by a final question, ‘What more opportunities to act do you have as a patient?’

The three situations were based on elements from real patient stories and previous studies as well as insights from interviews with two members of staff and two patients at the host clinic for the intervention. The three situations were created to be different from each other and built on what can be called *archetypical* elements of abuse that have figured in Swedish studies [12, 15, 16]: blaming and offending a patient, not taking patients seriously, and making patients feel invisible. The situations are also different with regard to how isolated the care encounter is and whether others witness the interactions. The three situations were the following:

1. *‘Being scolded.’* A young patient without specific gender attributes enters a room where a middle-aged male health professional greets with his back turned toward the patient. The health professional then asks whether the patient performed the provided exercises, to which the patient answers, ‘I haven’t done them, because…’ The professional gets angry and starts yelling, wondering why the patient has not done the exercises, and complains about his or her laziness, while the patient tries to give an explanation in vain.
2. *‘It hurts!’* A young male patient lies in a hospital bed with a broken leg, surrounded by a few other patients and staff. A female health professional enters the room and says it is time to examine the patient’s leg. The examination is apparently painful, and while the professional continues to examine the patient’s leg, the patient says it is really painful, to which the professional responds, ‘It’s not that bad – we’ll be done soon.’
3. *‘A phone call.’* A female health professional is about to perform a pelvic exam on an elderly woman seated in a gynecology chair with her legs in the stirrups. An assistant female health professional stands next to the patient. After she has started, the examiner receives a phone call, which she immediately answers while remaining seated between the patient’s legs. The phone call is work-related, but apparently not of an emergency character.

These situations and the suggested spectrum of potential actions need to be understood as embedded in a Swedish health care context. In Sweden, health care is publicly financed and cornerstones of the health care system are equity of care and respectful treatment [26]. Rather recent developments in Sweden, as in many other western countries, include patients’ increased freedom of choice of providers informed by new public management [27] and different expressions of a push for more active patients, such as increased patient participation [28].

### Theoretical framework

The current study is situated in a theoretical framework of power, simply defined as the ability to act, as developed by feminist and critical theory scholar Amy Allen [29]. In her work she has aimed to integrate a wide range of theories of power (including those of Foucault, Butler, Lukes, and Arendt) in order to provide an approach that makes sense of complex power relationships. These relationships are in their turn a precondition for agency, the capacity to act [30] that relates to the self and the construction of identity [2].

Allen integrates three forms of power, which she states others have applied all too one-sidedly. The first is *power-over*, which focuses on domination; in other words, these are instances of unjust power over, and the non-trivial constraint of, others’ choices. The second is *power-to*, which centers on empowerment and the understanding of power regardless of, or in response to, others’ domination. The third is *power-with*, which attends to solidarity and acting collectively to attain a common end.

In many instances, abuse of patients has been analyzed as being cases of illegitimate power-over, or domination [8, 31, 32]. It is important to note that, in line with empirical research about abuse in health care, Allen [29] notes that instances of domination should be seen as embedded in most often unconsidered behaviors, without reference to intentions. This should instead shift the attention to structural power relationships that can be to the disadvantage of patients. Being interested in patients’ account of their potential to act in a subordinated and vulnerable position is, however, poorly understood from such a perspective, and is better seen as a form of *power-to*. While empowerment focuses on the potential to act in spite of domination, resistance consists of specific instances of empowerment that serve to subvert domination [29]. In line with Boal’s pedagogy on which the comics were based, the current study understands patients’ accounts of agency as instances of power as resistance in situations where they risk experiencing abuse.

## Method

### Design

We applied a qualitative study design using the methodology of constructivist grounded theory [33, 34]. Constructivist grounded theory assumes several core principles and methods from the original grounded theory [35] such as simultaneous data collection and analysis, theoretical sampling, and comparative analysis, although it emphasizes a constructivist epistemology in which results are viewed as co-constructed by researchers in a specific social setting [36]. In contrast to original grounded theory, it positions these principles and methods as a flexible *tool-box* rather than a fixed set of requirements [34], meaning that we used relevant tools that applied to our study, as described below. In contrast to a quantitative design, our aim is not to develop statistically generalizable results, but rather analyze patient stories and variations within these in order to explore and refine possible understandings and ideas related to patient agency.

### Data collection

Between October 2013 and January 2014 patients visiting a nephrology clinic in the south of Sweden were invited to participate in a larger online intervention study [21]. The project was connected to this particular clinic through a regional research coordinator, who had knowledge about the clinic’s willingness to engage in a project focusing on patient care. At the end of the intervention program participants were asked whether they were interested in being contacted about the current interview study, to which 26 (out of 48) replied positively. The sampling process started off as an open sampling aimed at maximizing variations in experiences and descriptions by using patients within the intervention program who had contrasting backgrounds and experiences. However, many potential informants could not be reached (*n* = 3), did not respond (*n* = 7), or declined participation (*n* = 3). This led to a shift towards convenience sampling, including all participants who were willing to take part in an interview. Ultimately, thirteen informants consented to participate in the interview study; the first author conducted the interviews between February and May 2014. Background characteristics of the informants are presented in Table 1.

**Table 1:** Background characteristics of the thirteen informants

Gender (n)	
Woman	6
Man	7
Age (range in yrs)	
	42–81
Highest education (n)	
Primary	1
Secondary	8
Higher	4
Reported experiences of abuse in health care according to the Norvold Abuse Questionnaire [47] (n)	
Yes	4
No	9

Knowing that many patients at this clinic are chronically ill and have frequent hospital visits, the participants were offered their choice of place for the interview. Two patients were interviewed during hemodialysis at the clinic, six in a conference room at the hospital, three at their workplace, and two in their homes. The interviews followed a semi-structured interview guide that was adjusted multiple times after the first couple of interviews, following preliminary analytical strands. The guide contained open-ended questions about abuse in health care; acting in abusive situations; and, for all three comics, about what happens and opportunities to act in the situation. On average the interviews lasted 51 min, ranging from 28 to 78 min. The interviews were digitally recorded and transcribed verbatim. The original interviews were conducted and analyzed in Swedish and relevant parts were translated for the purpose of this article.

### Data analysis

In line with a constructivist approach [34], the coding process was not conducted with an empty head but rather an open mind, in which our different scholarly backgrounds were used as analytical starting points in order to open up, understand, and problematize data [37]. Our backgrounds are in medical sociology (first author), and social psychology and educational research (second and third author). During the analysis, we followed Charmaz’s [34] guidelines for coding in constructivist grounded theory studies. The coding process was divided into four parts: (i) preliminary memo’s (ii) initial coding, (iii) focused coding, and (iv) theoretical coding. First, the first author wrote preliminary memo’s after each interview, reflecting on possible analytical strands thereby allowing data collection, through revision of the interview guide, to follow and address these insights. In this way, data collection and analysis were to some extent conducted simultaneously [34]. Second, we analyzed the interviews line-by-line in a way that was fast, provisional, and close to the data, guided by a set of analytic questions such as what is going on here and what is the informant’s main concern(s). Third, during focused coding, we synthesized the most significant initial codes and conceptualized them into categories in order to cover larger parts of the data. Especially during this part of the analysis and onward, extensive memos were written containing analytical reflections. During this stage we could conclude that the material as a whole contained a rich amount of detail and variation in positions and perspectives in order to construct a nuanced understanding of patient agency; signs of rich data, despite a small group of participants [34]. Fourth, in theoretical coding, the larger clusters of data that were formed into categories during focused coding, and their relationships to each other, were integrated into a grounded theory of patient agency in health care situations of abuse. We continued refining the analysis until we reached theoretical saturation in all categories, meaning further analysis and re-analysis did not reveal new relevant insights [34]; the categories and their relations appeared to be robust. The entire coding process was performed in a non-linear fashion going back and forth between codes and data, and between different levels of coding.

### Ethics

The study was approved by the Regional Ethical Review Board in Linköping, Sweden (reg.no. 2013/242–31). Participants received written information about the study by email upon invitation, and received similar written and verbal information before the start of the interview. Both the participant and the interviewer (the first author) signed an informed consent form that emphasized that participation was voluntary and that participants could withdraw at any time. Participants were assured that all research material would be treated confidentially.

## Results

The informants’ stories concerning their potential to act in abusive situations, either experienced ones or imagined ones such as in the comics, showed that they talked about a patient’s agency as something elastic and negotiable in specific situations. General assumptions and situation-specific conditions were not only intertwined, but also re-defined during reflections. *Re-negotiating agency* runs as a core process through the different domains of consideration below, signifying that the informants’ reflections were far from fixed and predetermined. For example, apparently small alterations in their interpretations of the comics could completely transform their reasoning about what is possible and what is not. We constructed three broad categories that elicited the different dimensions the informants included in negotiations of their agency: re-defining the event, re-defining responsibilities, and re-considering values and needs.

### Re-defining the event

Re-defining the event refers to how the informants defined the situation they encountered and whether it was interpreted as an abusive situation or not. These definitions affected their potential to act. In general, all informants agreed that abuse was wrong and something that should demand patients’ agency.

I also want to stress that the responsibility you manage to take, have the sense to take, and the knowledge to take, that is what you should take action on, in the relationship–, in your relationship with the caregiver. I would never accept someone mistreating me. (Informant 2)

Despite this agreement, their definitions varied, with many informants concerned with analyzing exactly what phenomenon they were asked about, and then talked about it in relation to the comics. Some found abuse to be too strong a word to describe the events pictured in the comics, and preferred to talk about such situations as being wrongful or disrespectful. Abuse, in their eyes, is a serious event, where patients feel grossly degraded and perhaps even deliberately harmed, not ‘just’ disrespectfully treated. The emphasis on definitions was especially common among informants who relayed that they had not experienced any abuse in health care. Interestingly, these informants had experienced events of a similar kind to the other informants, who did talk about them as abusive, but the events never threatened their own dignity or identity. Consider the following excerpt where an informant talks about the staff’s actions as problematic rather than abusive or humiliating.

Just because they prioritize the phone over the one they have in front of them, I don’t feel of less value because of that. Instead, I think that [member of staff] is just making a bad judgment call. (Informant 3)

Not defining an incident as abuse could either open up for a certain potential to act, or lead to passivity, depending on the extent to which an informant found a situation wrongful. Sometimes the informants also made distinctions between being too sensitive and being abused, which revealed a further problem with defining the concept as well as highlighting how some patients were defined as ‘too sensitive’ and as complaining ‘too much’.

Among the interpretations of the different situations represented in the comics, some patients did not see every situation as problematic. For example, in ‘It hurts!’ a variety of interpretations were seen. Some patients focused on the dismissal of the patient addressing his pain, while others accepted that pain was somehow inevitable in the situation, or instead stressed that the patient was poorly informed. This comic, more than the others, shows that the extent to which patients define this situation as abusive or even problematic relates to more general ideas of the status and necessity of pain. One informant related it to a painful experience where a doctor had problems suturing the patient’s leg.

Actually, if it hurts that bad you should–, if it was me, I would have said, “Listen, you have to stop with that and give me some anesthesia.” But it’s a bit difficult to say that in such a situation. [Interviewer: What is it that makes it difficult, do you think?] It’s a quick thing, they pull three threads and it happens so fast. So perhaps you think that you can tolerate it. (Informant 12)

Related to this, one informant stressed that although he could see a problem in most of the comics, which could make him feel frustrated rather than abused, he would nevertheless try to tolerate caregivers’ mistakes. This implied that, for him, there was no great need to look for potential to act, but rather to accept things as they are and not feel bad about it. Reflecting on ‘It hurts!’ he stated:

Were this to happen, I would rather feel angry than [*laughs*] abused. And angry doesn’t mean that I’d start to swear, but afterwards I would just [*laughs*] state that this was a failed procedure, or that it was unnecessary. (Informant 13)

Different types of abuse open up for different potentials for action. In ‘A phone call’ there were two factors, or a combination of them, that informants identified as important: (i) the woman’s exposed position, and (ii) the fact that a non-urgent phone call was prioritized over the woman. Both options can, however, lead to the same type of response, through different types of reasoning.

I would have gotten up and left the room. [Interviewer: When? At what point?] Right when she answers the phone. Especially in such a situation when you can see that it’s a gynecology chair – you’re really exposed. (Informant 5)

It’s like when we get to the grocery store and you explain which kind of milk you’d like, and they answer a phone call. Then, I’d leave – we’re done talking, because that person just showed me that he or she cares less about me than their phone. (Informant 4)

The first informant stresses the fact that the patient is very exposed and sees leaving as a straight-forward strategy for immediate self-protection. The second informant also talks about leaving a situation, but rather emphasizes not being prioritized.

### Re-defining responsibilities

A second concern for the informants was their view on responsibilities and roles; this included general assumptions about patient roles and patients’ responsibilities toward themselves and toward staff and other patients. However, responsibilities were re-defined according to the situation. Interestingly, before reflecting on patients’ responsibilities and agency, in most instances the informants almost immediately responded to the comics by pointing out what the staff in the situation could or should have done, thereby instead emphasizing the staff’s responsibilities in the situation.

While staff to some extent was subscribed as those responsible for good care, their responsibilities were not viewed as absolute since the informants also addressed staff’s behaviors as occurring within structural constrains. Through this, some of staff’s actions were more understandable and therefore less perceived as a threat toward a patient. Similar lines of reasoning concern the caregiver’s personal situation, as one of the informants discussed in relation to ‘Being scolded’:

I can also think like, what has happened to him that morning, and so on. [Interviewer: What has happened to the caregiver?] Yes, the caregiver. A stressful situation, or does he have a lot to do, and yes, I mean he’s more dissatisfied with his own situation than with the person in question. (Informant 10)

Analyzing the staff’s situation and their room for agency could cast some responsibility back to the patient for making staff aware of their behavior, for aiding staff in making changes and thereby protecting future patients, which the informants defined as part of their responsibility too.

There appeared to be a general relationship between the informants’ efforts to understand the staff’s problematic situation and the tendency to perceive a situation as abusive. Informants who themselves had not reported any experiences of abuse in health care seemed more likely to engage in efforts through which they could understand the staff’s problematic situation. Thinking about patients’ roles and options, although talked about in a positive and imperative way, was not always obvious. Talking about patient responsibilities started very generally, with patients being responsible for informing themselves and having knowledge about their own bodies. This knowledge would then have a preventive function and could protect oneself from abusive encounters, as well as also make patients active and prepared.

As a patient you should learn that when things are not okay, learn to speak up. Make clear that this hurts today, and can I get help with that. (Informant 7)

Another aspect of this addressed a division between a new and a traditional patient role that was interlinked with a view on a changed health care system that subscribed to the patient being an equal part and as someone who has agency.

I think it’s–, I can see it among older people that I know around me. Doctors used to have a high status in society before, like priests or the bourgeois; someone that you look up to. My present doctor, I don’t look up to him more than I value his great knowledge that I need. And then I respect him for his knowledge, but I don’t see him as a higher power. (Informant 4)

The general assumption of the active patient was re-negotiated in specific encounters where it was balanced against conditions that could affect patient roles and responsibilities. These conditions were closely related to patients’ vulnerability and status, and could reduce patients’ agency. The informants seemed to be caught between positioning patients as responsible to act, yet at the same time they made distinctions between different patients as more or less responsible due to their own capabilities. It was commonly found that the informants viewed their own room for agency as larger by positioning themselves against more vulnerable patients. Vulnerability was related to sickness, age and social status.

I think it differs a lot. Now I still have the energy and can assert myself quite well. I think I stand up for myself pretty well so far, but eventually, the older you become, the worse it gets. (Informant 11)

Interestingly, the oldest informant, an 81 year old man, confirms the importance of capabilities, but relates this to stamina and health, rather than age.

I think it depends on how sick you are, how much the patient manages to stand up against such things. (Informant 12)

It is interesting to note that some of the informants point out that later in life, or when they get too sick to act, that they might lose some of their agency. Thus they view their agency as negotiable and changeable throughout a lifetime, rather than a fixed trait or possession.

### Re-considering values and needs

The third account of importance for the informants’ agency was articulated as aspects of values and needs. This refers to the informants’ assumptions about the perceived intrinsic value of the health care system – an environment based on trust, respect and warm social relationships – that were further elaborated on as the ultimate ends for any form of patient agency. An important narrative was that the patients’ actions should not threaten these intrinsic values, or the social relationships to their caregiver or the health institution. Not damaging the relations to a caregiver was also of instrumental value, as the informants stressed that acting in any way may entail a risk of not getting necessary care. In response to acting in ‘A phone call’:

Well, it’s not going to improve my situation in any way. On the contrary, I risk getting even more ill-treated. […] No, I wouldn’t do that, never in my life, as a patient. I’m there because of a reason – that I’m not feeling well and I want to get treated. No, I wouldn’t do it. (Informant 11)

Patient actions are motivated by several needs, either to control a situation and protect oneself, to prevent future incidents, or to vent frustration, but these are contrasted against risks of provocation and escalation.

Well, you can fight in a wise way, without wasting any negative energy and shouting and yelling and so on, because I think you have better control of the situation, over all the details in the situation, so you can be clear and receive help. (Informant 6)

This informant displays a form of tactical reasoning, thinking of actions that would be constructive for the encounter and trying to anticipate the staff’s reaction to certain actions. Provoking a caregiver can make a situation worse, with the patient running the risk of not receiving essential care, which seems to function as a sort of bottom line for the range of actions that seem feasible. At the same time, the informants acknowledge a need for actions that are directed at the professional in question. Possibilities such as including other staff or patients were not seen as valuable options, and could provoke the professional in question. But there is more to this. Informants also show signs of an inherent logic of confronting the professional who displayed abusive behavior. About ‘It hurts!’ an informant says:

I wouldn’t talk to other staff. No, I’d turn directly to the one who’s doing things, I think. It’s weird to talk to someone else when she’s right there in the room. (Informant 8)

Some also viewed direct confrontation as a better alternative to indirect strategies, such as contacting the clinic or hospital management. This may be associated with the fact that it can be of great value for the patient if the professional in question confirms the patient’s feelings.

How you’re treated, it’s a lot about that – if you should go further with this. If the professional solves this, well depending on how serious the incident was, but I mean if you honestly feel that that person says, “Oh, yes, that probably wasn’t so good, I’m sorry, I didn’t think of it,” then I would probably be happy with that. (Informant 8)

Such a confirmation might mean more to patients than recognition from other staff or a committee. That other staff can be valuable, and a part of a patient’s room for agency was mostly visible in situations where informants found direct confrontation too risky or intimidating. So, rather than involving other staff in a situation, they would talk to them afterwards. One informant explained that she would not dare to confront a doctor, and would rather accept what happens and then complain to a nurse afterwards. However, there was also the view of patients as customers that seemed to strengthen their agency:

[i]f I go to the store and want to buy a special kind of milk and they say, “We’ll never provide that milk in our assortment,” then I’ll go to another store that can provide my milk. And if they act like that toward me, I’ll switch doctors. That’s just the way it is. (Informant 4)

Throughout the results, we have discussed how *re-negotiating agency* ran as a core process through the informants’ considerations, exemplifying, according to the three broad categories identified, how their agency was ongoing rather than static.

## Discussion

This study aimed to elicit patients’ reflections upon potential to act in situations of abuse, as displayed in a series of comics, and found that agency was constantly re-negotiated depending on the situation, specific responsibilities, and relevant values and needs. The negotiation processes elicited in the informants’ reflections problematize any ideas of patient agency as predetermined and self-evident.

Among the informants there seemed to be an ongoing positioning of who has the responsibility to do something about abuse in health care. Overtly, they view health care as responsible for doing something or keeping these abusive events from ever occurring by fulfilling their social role as *caregivers*. At the same time, patients were defined as having agency, even if obstacles that limit their room for agency were quickly addressed. Oftentimes this was by stating that the informants themselves have agency; however, older patients, very sick patients, or non-informed patients have limited agency. Some informants also positioned themselves in contrast to the abused patient. Among some of the informants there was a tendency to address that nowadays some people complain about everything, even though they, according to how some informants defined it, are *not really* abused. They also noted that sometimes you have to have a certain tolerance level for wrongs and clumsiness. This re-defining the event by softening the blow could demotivate patients from taking action, because there was no perceived abuse to act upon. This might be interpreted as a form of identity work, resisting defining oneself as a victim, but at the same time reveals that informants were occupied with trying to define what is abusive and what is not. Defining abuse mattered, and this study is not the first to show that it matters. In a qualitative study about staff perceptions of abuse in health care, the informants heavily engaged in describing a variety of aspects related to abuse [14]. Similar to the informants in our study, these staff informants also talked about responsibilities, and several ways to avoid them, including stating that abuse can happen at any time, that all patients are different, and that some patients just may be prone to label certain events as abusive. Interestingly, the latter aspect is similar to expressed skepticism toward other patients’ claims of being abused in the current study.

While focus in our interviews was on instances of resistance or self-protection by asking informants to reflect upon their potential to act in abusive situations, they were concerned with other forms of agency as well. Preparing oneself before a health care encounter by getting informed, knowing one’s needs, and perhaps even having a strategic plan about how to navigate in a complex health care system, can be poorly understood as instances of resistance. Instead, this can be seen as what Allen [29] calls *empowerment* by referring to agency in spite of, rather than in response to, domination. Yet another form of agency could be seen in informants’ focus on acting to improve care for future patients. This can be much better understood as what Allen calls *solidarity*, in other words, acting for a common purpose, rather than protecting oneself. In her review of battered women’s protective strategies, Sherry Hamby [38] describes similar strategies when looking at women’s stories and how they act to protect themselves. What she calls *immediate situational strategies*, are strategies for self-protection in direct situations of assault. Outside of these immediate actions for self-protection, other protective strategies include formal help-seeking and protecting others (e.g., children). Another interesting group are the *invisible strategies*, those that for several reasons have been ignored by researchers, but show the women’s creativity in their heavily confined action space. In the current study, in line with how the comics were constructed, we focused on immediate situational strategies, although our findings aroused an interest in analyzing strategies outside of the abusive situation itself: What do patients do to organize their care in ways that protect themselves (and others) in the long run? Studying such questions, instead of focusing on patients’ helplessness would be in line with an ongoing trend in violence research with the focus on victims’ creativity, strengths and resilience [38]. This could also be an analytical entry into understanding what patients identify as threats and barriers to the health care they need, and how these can be tackled.

The re-negotiation of agency may reveal an ongoing positioning of the social role of the patient in larger discourses about patients’ possibilities to shape their care. In many ways, the informants addressed new roles in the ongoing marketization of the Swedish health care system, where patients are active customers. This new role has different implications depending on whether patients’ voice or patients’ exit strategies are emphasized [39], and in the current study, the informants discussed both strategies (both were also included in the comics). Hirschman [40] defined voice strategies as all activities through which customers can express their concern to a provider, while exit strategies consist of actions to leave a provider. Voicing activities usually demand more creativity and effort compared to straightforwardly leaving a provider [40], and yet many informants presented voicing activities as most reasonable and constructive in most situations. Elements that could stop our informants from using voicing activities would be when they perceive there is a risk of not getting the necessary treatment, or that the caregiver would feel provoked. Otherwise, voicing was seen as most constructive in terms of immediately finding a way out of the situation, exercising self-protection, as well as offering caregivers a learning opportunity. Whether the patient’s action turns into a learning opportunity depends on whether and how staff respond to this action [39, 41].

Some limitations of this study should be noted. Firstly, the study’s clinical setting needs to be considered, as we only recruited informants through one nephrology clinic. But rather than discussing this as a limitation, we introduced nephrology as a context to the interviews and the analysis – the research aims were not particular to nephrology. All the informants had long-term experiences and frequent encounters with the health care system, which may be relevant to some reflections on agency that relate to long term care and re-visiting a caregiver. But, and this is in line with earlier interview studies on the topic [32], most of the situations, examples, and experiences come from other health care settings, not necessarily from within nephrology settings, creating diversity in the material. Secondly, all our informants were Swedish speaking, over the age of forty, and with often long-term and frequent health care contact. Our analyses should be seen against this background, as verbal strategies, age and health status all relate to patients’ agency. Thirdly, our study elicits the informants’ reasoning about agency based on their verbal responses to hypothetical scenarios of abuse in health care. How the participants respond to hypothetical scenarios and what they said they think and do are not the same as investigating how they respond in real-life situations. The ecological validity is therefore threatened [42]. However, this approach enabled us to collect reflections from all the participants about the ‘same’ situations, although all informants noticed different aspects in the situations and identified with the characters differently. This was likely based on, for example, the characters’ complaints, age, genders, but also how the informants historically have been exposed to and responded to related situations [43]. While some developmental psychological studies have demonstrated that the judgments participants make of transgressions in actual situations generally correspond with their judgments of transgressions in hypothetical situations [44, 45], which addresses the issue of ecological validity, it is likely that the patient’s agency is negotiated differently when actually being exposed to abuse in health care. For that reason, ethnographic work and case studies would be worthwhile to pursue in further research. Fourthly, because all interviews were conducted before we began the initial line-by-line coding, we were unable to take full advantage of theoretical sampling [34, 35] as a guiding principle in our data collection. Despite this limitation, we were still able to reach theoretical saturation, indicating that the collected data were sufficiently rich in depth and variation. However, theoretical sampling as an iterative process between data collection and analysis might have strengthened and further elaborated our findings. Finally, the small and non-probability sample limits the transferability of the findings. Nevertheless, instead of statistical generalization built upon the logic of mathematics, in qualitative research, generalization has been discussed as an interpretation work – for example, in terms of generalization through recognition of patterns, in which the reader, not the researcher, judges generalizability [46]. In line with a constructivist grounded theory tradition, we do not claim to offer an exact picture but rather an interpretative portrayal of the phenomenon studied [34].

## Conclusion

This study uniquely explores patients’ reasoning about their potential to act in situations where they risk experiencing abuse. The three comics that were used as vignettes elicited a process of constant re-negotiation of the patients’ agency in relation to specifics of the situation, the involved persons’ responsibilities, and relevant values and needs. Not only did the informants explore immediate actions in response to a situation, they also discussed less visible strategies that could protect them and counteract abuse in health care over the long-term. This insight questions the idea of patients’ silence toward the health care system in relation to abusive encounters, as described in our Background, as a form of acceptance or passivity. Rather it opens up for studies into ways patients organize their care and how they navigate against and within health care structures. For the health care system, this type of knowledge may be important in addition to a focus on voice and exit activities that now are assumed to drive quality improvement [39].

## References

[1] Burkitt I (2016). Relational agency: relational sociology, agency and interaction. Eur J Soc Theory.

[2] Cussins C (1996). Ontological choreography: agency through objectification in infertility clinics. Soc Stud Sci.

[3] Bandura A (2006). Toward a psychology of human agency. Perspect Psychol Sci.

[4] Swahnberg K, Hearn J, Wijma B (2009). Prevalence of perceived experiences of emotional, physical, sexual, and health care abuse in a Swedish male patient sample. Violence Vict.

[5] Swahnberg K, Schei B, Hilden M, Halmesmaki E, Sidenius K, Steingrimsdottir T (2007). Patients' experiences of abuse in health care: a Nordic study on prevalence and associated factors in gynecological patients. Acta Obstet Gynecol Scand.

[6] Coyle J (1999). Exploring the meaning of ‘dissatisfaction’ with health care: the importance of ‘personal identity threat’. Sociol Health Illn.

[7] d'Oliveira AFPL, Diniz SG, Schraiber LB (2002). Violence against women in health-care institutions: an emerging problem. Lancet.

[8] Stevens PE (1996). Lesbians and doctors: experiences of solidarity and domination in health care settings. Gend Soc.

[9] Chadwick RJ, Cooper D, Harries J (2014). Narratives of distress about birth in south African public maternity settings: a qualitative study. Midwifery.

[10] Jewkes R, Abrahams N, Mvo Z (1998). Why do nurses abuse patients? Reflections from south African obstetric services. Soc Sci Med.

[11] Lukasse M, Schroll AM, Karro H, Schei B, Steingrimsdottir T, Van Parys AS (2015). Prevalence of experienced abuse in healthcare and associated obstetric characteristics in six European countries. Acta Obstet Gynecol Scand.

[12] Brüggemann AJ, Wijma B, Swahnberg K (2012). Abuse in health care: a concept analysis. Scand J Caring Sci.

[13] Schroll A-M, Kjærgaard H, Midtgaard J (2013). Encountering abuse in health care; lifetime experiences in postnatal women-a qualitative study. BMC Pregnancy Childbirth.

[14] Swahnberg K, Zbikowski A, Wijma B (2010). Ethical lapses: staff’s perception of abuse in health care. J Psychosom Obstet Gynaecol.

[15] Swahnberg K, Thapar-Björkert S, Berterö C (2007). Nullified: women’s perceptions of being abused in health care. J Psychosom Obstet Gynaecol.

[16] Swahnberg K, Wijma B, Hearn J, Thapar-Björkert S, Berterö C (2009). Mentally pinioned: men's perceptions of being abused in health care. Int J Mens Health.

[17] Speraw S (2009). “Talk to me—I’m human”: the story of a girl, her personhood, and the failures of health care. Qual Health Res.

[18] Mulcahy L, Tritter JQ (1998). Pathways, pyramids and icebergs? Mapping the links between dissatisfaction and complaints. Sociol Health Illn..

[19] Wijma B, Thapar-Björkert S, Hammarström NC, Swahnberg K (2007). Cycles of abuse nurtured by concealment: a clinical report. J Psychosom Obstet Gynaecol.

[20] Brüggemann AJ, Wijma B, Swahnberg K (2012). Patients’ silence following healthcare staff's ethical transgressions. Nurs Ethics.

[21] Brüggemann AJ, Swahnberg K, Wijma B (2015). A first online intervention to increase patients’ perceived ability to act in situations of abuse in health care: reports of a Swedish pre-post study. BMC Med Ethics.

[22] Green MJ, Myers KR (2010). Graphic medicine: use of comics in medical education and patient care. BMJ.

[23] Boal A (2000). The rainbow of desire: the Boal method of theatre and therapy.

[24] Boal A (2000). Theatre of the oppressed.

[25] Österlind E (2008). Acting out of habits–can theatre of the oppressed promote change? Boal's theatre methods in relation to Bourdieu's concept of habitus. Res Drama Educ.

[26] Ministry of Health and Social Affairs. Hälso och Sjukvårdslag (Health and Medical Services Act [2017:30]). Stockholm, Sweden: Ministry of Health and Social Affairs; 2017.

[27] Fredriksson M (2013). Is patient choice democratizing Swedish primary care?. Health Policy.

[28] Angel S, Frederiksen KN (2015). Challenges in achieving patient participation: a review of how patient participation is addressed in empirical studies. Int J Nurs Stud.

[29] Allen A (1998). Rethinking power. Hypatia.

[30] Allen A (2002). Power, subjectivity, and agency: between Arendt and Foucault. Int J Philos Stud.

[31] Barber CF (2007). Abuse by care professionals. Part 3: analysis of characteristics and reasons. Br J Nurs.

[32] Brüggemann AJ, Swahnberg K (2013). What contributes to abuse in health care? A grounded theory of female patients’ stories. Int J Nurs Stud.

[33] Charmaz K, Morse JM, Stern PN, Corbin JM, Bowers B, Charmaz K, Clark AE (2009). Shifting the grounds: constructivist grounded theory methods. Developing grounded theory: the second generation.

[34] Charmaz K (2014). Constructing grounded theory.

[35] Glaser B, Strauss A (1967). The discovery of grounded theory: strategies for qualitative research.

[36] Thornberg R, Charmaz K, Flick U (2014). Grounded theory and theoretical coding. The SAGE handbook of qualitative data analysis.

[37] Thornberg R (2012). Informed grounded theory. Scand J Educ Res.

[38] Hamby S (2014). Battered women’s protective strategies: stronger than you know.

[39] Brüggemann AJ (2017). Exploring patient strategies in response to untoward healthcare encounters. Nurs Ethics.

[40] Hirschman AO (1970). Exit, voice, and loyalty: responses to decline in firms, organizations, and states.

[41] Wijma B, Zbikowski A, Brüggemann AJ (2016). Silence, shame and abuse in health care: theoretical development on basis of an intervention project among staff. BMC Med Educ.

[42] Cicourel AV (1982). Interviews, surveys, and the problem of ecological validity. Am Sociol.

[43] Sundel M, Sundel SS (2005). Behavior change in the human services: behavioral and cognitive principles and applications.

[44] Smetana JG, Schlagman N, Adams PW (1993). Preschool children's judgments about hypothetical and actual transgressions. Child Dev.

[45] Turiel E (2008). Thought about actions in social domains: morality, social conventions, and social interactions. Cogn Dev.

[46] Larsson S (2009). A pluralist view of generalization in qualitative research. Int J Res Meth Educ.

[47] Swahnberg K, Wijma B (2003). The NorVold abuse questionnaire (NorAQ): validation of new measures of emotional, physical, and sexual abuse, and abuse in the health care system among women. Eur J Pub Health.

